# Surgical outcomes and optimal approach to treatment of aortic valve endocarditis with aortic root abscess – systematic review and meta-analysis

**DOI:** 10.1177/02676591221137484

**Published:** 2022-10-31

**Authors:** William M Harris, Shubhra Sinha, Massimo Caputo, Gianni D Angelini, Hunaid A Vohra

**Affiliations:** Bristol Heart Institute, 1980 University of Bristol, Bristol, UK

**Keywords:** infective endocarditis, aortic root abscess, patch reconstruction, aortic root replacement, surgical outcomes

## Abstract

**Background:**

Data on the postoperative outcomes for patients with infective endocarditis complicated by an aortic root abscess is sparse due to the condition’s low incidence and high mortality rates. This systematic review and meta-analysis aims to evaluate existing data on the impact of aortic root abscesses on the postoperative outcomes and to inform optimal surgical approach.

**Methods:**

The online databases MEDLINE, EMBASE and Cochrane library were searched from 1990 to 2022 for studies comparing cohorts of surgically managed infective endocarditis patients with and without an aortic root abscess. Data was extracted by two independent investigators and aggregated in a random-effects model. Risk of bias was assessed using an adapted version of the Newcastle-Ottawa scale.

**Results:**

Six clinical studies were included in the meta-analysis (*n* 1982). The abscess group was associated with increased in-hospital mortality (OR 1.74 95%: CI 1.18–2.56) and late mortality (HR 1.27 95% CI:1.03–1.58). The reoperation meta-analysis was complicated by high rates of heterogeneity (I^2^ = 59%) and found no significant differences in reoperation between abscess and no abscess groups (HR=1.48: 95% CI:0.92–2.40). Post-hoc scatter graph showed a strong linear relationship (r 0.998), suggesting hospitals with higher rates of aortic root replacement achieve lower rates of reoperation for aortic root abscess patients compared with patch reconstruction.

**Conclusions:**

The presence of an aortic root abscess in aortic valve endocarditis is associated with elevated early and late mortality despite modern standards of care. Additionally, aortic root replacement should be considered to have a favourable postoperative profile for use in this context.

## Introduction

Approximately half of all infective endocarditis (IE) patients are identified as high-risk and undergo operative treatment.^1^ The presence of a paravalvular abscess is a crucial indication for surgical management^2^ due to its association with increased in-hospital mortality.^3^ However, the aetiology, sequelae and management of patients with paravalvular abscesses occurring in heart regions are distinct.^4^

Aortic root abscesses (ARA) are the most common type of paravalvular abscess,^5–7^ previously described as a catastrophic complication of IE. If left untreated, ARA can cause severe valvular dysfunction, heart block, pseudoaneurysm formation and obstruction to coronary blood flow.^8,9^ However, the impact of an ARA on early and late postoperative outcomes is currently not well defined given modern treatment standards. This is a consequence of the conditions’ low incidence and high mortality rate alongside the emergence of modern surgical techniques. Therefore, the evidence base on the postoperative outcomes of ARA consists of a fragmented collection of retrospective cohort studies.^5^

The two most common approaches to the treatment of ARA are patch reconstruction (PR) and aortic root replacement (ARR). Additionally, the aortic annulus can be repaired using interrupted sutures. The choice between these approaches is currently based on the extent of infection, surgeon or institutional preference and demographic factors.^9^ Although expert opinion will remain crucial in choosing the surgical approach required for each patient, as more data emerges, we should consider how medical literature can inform this decision.^10^

In this systematic review and meta-analysis, we aim to establish the significance of ARA on key postoperative outcomes and consider how this can help inform surgical decision-making between PR and ARR.

## Methods

The protocol for this review is registered on the PROSPERO website, registration number: CRD42021249932. This study used guidelines set out by the Prescribed Reporting Items in Systematic Reviews and Meta-analyses (PRISMA).

### Literature search strategy

A literature search was run to capture all published studies between 1990 and 2022 that have directly compared the postoperative outcomes of IE patients with and without an ARA. Using the keyword terms ‘infective endocarditis’, ‘aortic root abscess’, ‘periannular abscess’, ‘perivalvular abscess’ and ‘paravalvular abscess’ we searched three medical databases: PubMed, Embase and Cochrane library (Table 1).

**Table 1.** Medline (Ovid) full search strategy.

Search number	Query	Search details	Results
**7**	(Infective endocarditis) AND ((((aortic root abscess) OR (periannular abscess)) OR (perivalvular abscess)) OR (paravalvular abscess))	(“endocarditis” [MeSH terms] OR “endocarditis” [All fields] OR (“infective” [All fields] AND “endocarditis” [All fields]) OR “infective endocarditis” [All fields]) AND (((“aorta” [MeSH terms] OR “aorta” [All fields] OR “aortic” [All fields] OR “aortics” [All fields]) AND (“plant roots” [MeSH terms] OR (“plant” [All fields] AND “roots” [All fields]) OR “plant roots” [All fields] OR “root” [All fields]) AND (“abscess” [MeSH terms] OR “abscess” [All fields] OR “abscesses” [All fields] OR “abscessation” [All fields] OR “abscessed” [All fields] OR “abscessing” [All fields])) OR (“periannular” [All fields] AND (“abscess” [MeSH terms] OR “abscess” [All fields] OR “abscesses” [All fields] OR “abscessation” [All fields] OR “abscessed” [All fields] OR “abscessing” [All fields])) OR (“perivalvular” [All fields] AND (“abscess” [MeSH terms] OR “abscess” [All fields] OR “abscesses” [All fields] OR “abscessation” [All fields] OR “abscessed” [All fields] OR “abscessing” [All fields])) OR (“paravalvular” [All fields] AND (“abscess” [MeSH terms] OR “abscess” [All fields] OR “abscesses” [All fields] OR “abscessation" [All fields] OR “abscessed” [All fields] OR “abscessing” [All fields])))	789
**6**	(((Aortic root abscess) OR (periannular abscess)) OR (perivalvular abscess)) OR (paravalvular abscess)	((“aorta” [MeSH terms] OR “aorta” [All fields] OR “aortic” [All fields] OR “aortics” [All fields]) AND (“plant roots” [MeSH terms] OR (“plant” [All fields] AND “roots” [All fields]) OR “plant roots” [All fields] OR “root” [All fields]) AND (“abscess” [MeSH terms] OR “abscess” [All fields] OR “abscesses” [All fields] OR “abscessation” [All fields] OR “abscessed” [All fields] OR “abscessing” [All fields])) OR (“periannular” [All fields] AND (“abscess” [MeSH terms] OR “abscess” [All fields] OR “abscesses” [All fields] OR “abscessation” [All fields] OR “abscessed” [All fields] OR “abscessing” [All fields])) OR (“perivalvular” [All fields] AND (“abscess” [MeSH terms] OR “abscess” [All fields] OR “abscesses” [All fields] OR “abscessation” [All fields] OR “abscessed” [All fields] OR “abscessing” [All fields])) OR (“paravalvular” [All fields] AND (“abscess" [MeSH terms] OR “abscess” [All fields] OR “abscesses” [All fields] OR “abscessation” [All fields] OR “abscessed” [All fields] OR “abscessing” [All fields]))	856
**5**	Paravalvular abscess	“paravalvular” [All fields] AND (“abscess” [MeSH terms] OR “abscess” [All fields] OR “abscesses” [All fields] OR “abscessation” [All fields] OR “abscessed” [All fields] OR “abscessing” [All fields])	196
**4**	Perivalvular abscess	“perivalvular” [All fields] AND (“abscess” [MeSH terms] OR “abscess” [All fields] OR “abscesses” [All fields] OR “abscessation” [All fields] OR “abscessed” [All fields] OR “abscessing” [All fields])	234
**3**	Periannular abscess	“periannular” [All fields] AND (“abscess” [MeSH terms] OR “abscess” [All fields] OR “abscesses” [All fields] OR “abscessation” [All fields] OR “abscessed" [All fields] OR “abscessing" [All fields])	100
**2**	Aortic root abscess	(“aorta” [MeSH terms] OR “aorta” [All fields] OR “aortic” [All fields] OR “aortics” [All fields]) AND (“plant roots” [MeSH terms] OR (“plant” [All fields] AND “roots” [All fields]) OR “plant roots” [All fields] OR “root” [All fields]) AND (“abscess” [MeSH terms] OR “abscess” [All fields] OR “abscesses” [All fields] OR “abscessation” [All fields] OR “abscessed” [All fields] OR “abscessing” [All fields])	459
**1**	Infective endocarditis	“endocarditis” [MeSH terms] OR “endocarditis” [All fields] OR (“infective” [All fields] AND “endocarditis” [All fields]) OR “infective endocarditis” [All fields]	41,636

### Selection criteria

We included all studies which compared pre-specified postoperative outcomes in patients with surgically managed active infective endocarditis with and without an ARA. Studies were excluded based on inappropriate comparison groups and inclusion of mitral, pulmonary, or tricuspid valve abscesses. Additionally, paediatric studies, non-English texts, case reports and editorials were excluded.

**Figure 1.**
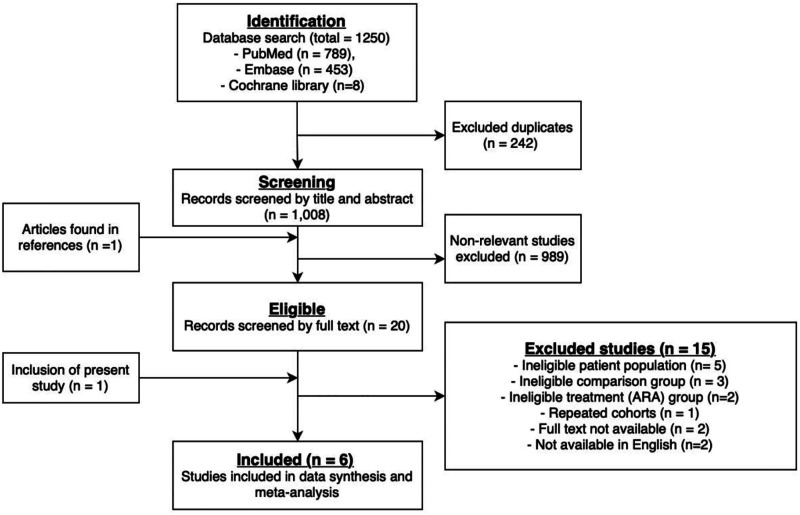
PRISMA review flow diagram demonstrating our systematic approach to study selection.

Two reviewers (WH, SS) performed independent dual screening of the search results, screened excluded articles, and verified data extraction. Discrepancies were resolved by consensus. A PRISMA flow chart of the search and study selection process is shown in Figure 1.

### Data extraction

A data collection sheet was used for study characteristics and outcome data. The following data was collected:

• Methods: Study design, recruitment method, follow-up period, date of the study, country of study and adjustments for confounding.
• Participants: Overall number, mean age, median follow-up, ARA incidence rate.
• Outcomes: In-hospital mortality, late mortality, and late reoperation.

Primary outcomes were in-hospital mortality (defined according to the Society of Thoracic Surgeons’ (USA) definition, including all causes of death occurring during the same hospitalisation in which the surgery was performed, even after 30 days^11^), late mortality and late reoperation. For outcomes late mortality and late reoperation, only studies which used a follow-up period of 5-years or longer were included.

### Statistical analysis

For our meta-analysis, a random-effects model was used due to anticipated heterogeneity. Where possible, adjusted odds ratios (OR) were included for in-hospital mortality and adjusted risk ratios (RR) for late outcomes. Heterogeneity was assessed using the I^2^ statistic and funnel plots were constructed to evaluate publication bias. Risk of bias was assessed using an adapted version of the Newcastle-Ottawa scale. Meta-analysis was performed using Review Manager (RevMan) [Computer program]. Version 5.4, The Cochrane Collaboration (UK), 2020.

### Assessment of in-study bias

Study bias was assessed using an adapted version of the Newcastle-Ottawa scale. Assessment of bias was performed in 7 domains:

1. Random sequence generation
2. Allocation concealment
3. Blinding of participants and personnel
4. Blinding of outcome measure
5. Incomplete outcome measure
6. Selective reporting
7. Other bias

Each domain was marked as having low bias (+), serious bias (−), or could not be assessed (?). The overall quality of evidence for each primary outcome was appraised using the GRADE scoring system.^12^

## Results

In total, our search of Medline, Embase and the Cochrane library yielded 1250 citations. After screening titles and abstracts, 20 studies were viewed to meet the inclusion criteria and were assessed for full eligibility (Figure 1).

Including our published study (Harris et al.^10^), six retrospective cohort studies were used in our meta-analysis, capturing 1982 cases of aortic valve IE and 518 (26%) cases of ARA. Studies were excluded based on; the use of a duplicated cohort, ineligible treatment or comparison groups and unavailable full texts written in English. Summaries of each study were tabulated (Table 2).

**Table 2.** Summary of studies included in our literature review and meta-analysis.

Reference (publication year)	Enrolment period	Country of study	Total cases	No ARA	ARA	ARA incidence, %	Mean age (years)	In-hospital mortality no ARA	In-hospital mortality ARA	Late follow-up provided	Reoperation data provided	Median follow up (months)	Other post-operative outcome	Recruitment method
Harris^10^ 2022	2009–2020	UK	143	93	50	35	58.5	7.5%	12.0%	Adjusted all-time	Adjusted all-time	43.0	NP	Surgically managed active aortic valve IE
Yang^17^ 2020	1996–2017	USA	336	157	179	53	55	3.8%	7.8%	Adjusted all-time	10-years cumulative incidence	45.6	Adjusted operative	IE patients treated with AVR or ARR
*Mahmoud* ^a^ ^15^ *2020*	NP	Egypt	165	121	44	27	31.5	24.8%	29.5%	NP	NP	81.6	NP	Left-sided IE*
Yoshioka ^13^ 2017	2009–2016	Japan	226	162	64	28	64	8.6%	17.2%	Unadjusted 5-years	NP	NP	NP	Surgically managed left-sided IE
Said^14^ 2017	1995–2013	USA	801	687	114	14	60	NP	NP	Adjusted all-time	Adjusted 30-days	57 (mean)	Adjusted 30-days	Surgically managed left-sided IE
Anguera^16^ 2005	NP	International merged database**	311	244	67	22	58	11.1%	19.4%	NP	NP	NP	NP	Surgically managed active aortic valve IE

Key – ARA, Aortic Root Abscess; NP, Not Provided.

^a^Mahmoud et al. study included all left-sided IE patients but only data from surgically managed IE patients was extracted from the study. Anguera et al. included data from institutions in Sabadell (Spain), Barcelona (Spain), North Carolina (USA), Philadelphia (USA), Besancon (France), Marseille (France), Goteborg (Sweden) and London (UK).

Recruitment methods varied between studies. The most common approach to data collection was the inclusion of all patients who received surgical management for left-sided infective endocarditis, identified from a local database and followed up using the electronic health record.^13,14^ Mahmoud et al. included medically managed patients in their study but only data associated with surgically managed patients with left sided disease was extracted for the purpose of this paper (Table 2).^15^ Using data from these three studies,^13-15^ the incidence rate among surgically managed left-sided IE patients was 18.6%. Other studies collected data by isolating all surgically managed patients with active aortic valve infection^10,16^ or based recruitment on the presence of an aortic valve or root operation in given IE infection.^17^

**Figure 2.**
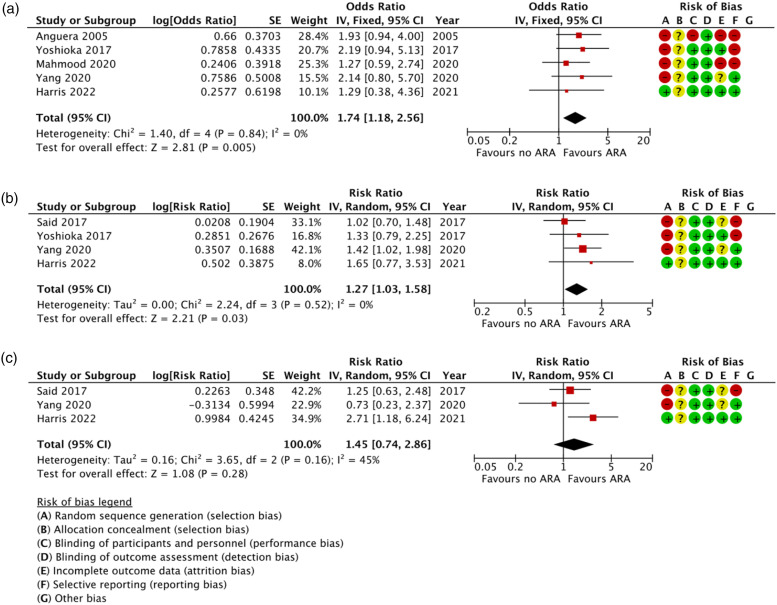
Meta-analysis forest plots for (a) in-hospital mortality, (b) late mortality and (c) late reoperation, comparing groups of patients with and without an aortic root abscess.

In-hospital mortality statistics were available in five out of the six clinical studies.^10,13–17^ Raw in-hospital mortality figures varied from 7.8% to 29.5% between institutions, with only one study^10^ adjusting their findings for confounding variables (Table 2). Despite the variation in raw mortality data, the variance of ORs heavily overlapped between studies leading to a low level of heterogeneity (I^2^ = 0%) (Figure 2(a)). Late mortality figures were available in four studies,^10,13,14,17^ with three of these using statistical models to adjust for confounding factors.^10,14,17^ Late mortality also had a low rate of heterogeneity (0%) (Figure 2(b)). Late reoperation rates were only available in three studies,^10,14,17^ with statistical adjustment present in two of these^10,14^ (Table 2).

Our meta-analysis yielded a significant association between ARA and increased risk of in-hospital mortality (OR = 1.74 95% CI: 1.18–2.56) (Figure 2(a)). Our meta-analysis also found that ARAs were also associated with a modest increase in long term mortality (HR = 1.27 95% CI: 1.03–1.58) (Figure 2(b)). However, our reoperation meta-analysis was inconclusive (HR = 1.48 95% CI: 0.92–2.49) and was subject to considerable heterogeneity (i^2^ = 45%) (Figure 2(c)).

**Figure 3.**
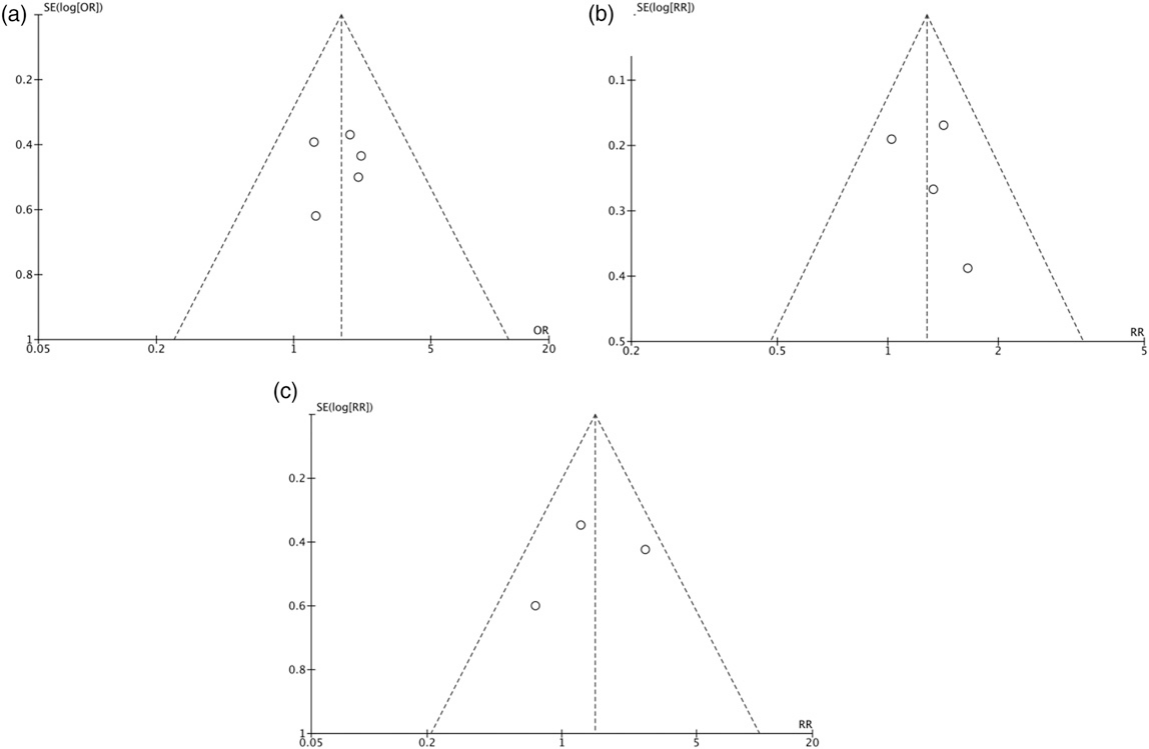
Meta-analysis funnel pots for (a) in-hospital mortality, (b) late mortality, and (c) late reoperation comparing groups of patients with and without an aortic root abscess.

Risk of bias is summarised in Figure 2. All studies were observational in nature, so risk of bias was inherently high. However, a systematic approach to data collection and the use of statistical models to adjust for confounding variables can reduce risk of bias.^10, 17^ These methods were not consistently used by articles included in our analysis; therefore the overall quality of the evidence was judged to be low according to the GRADE criteria.^12^ Funnel plots for each primary outcome suggested no substantial evidence of publication bias (Figure 3).

### Ecological analysis of reoperation rates

**Figure 4.**
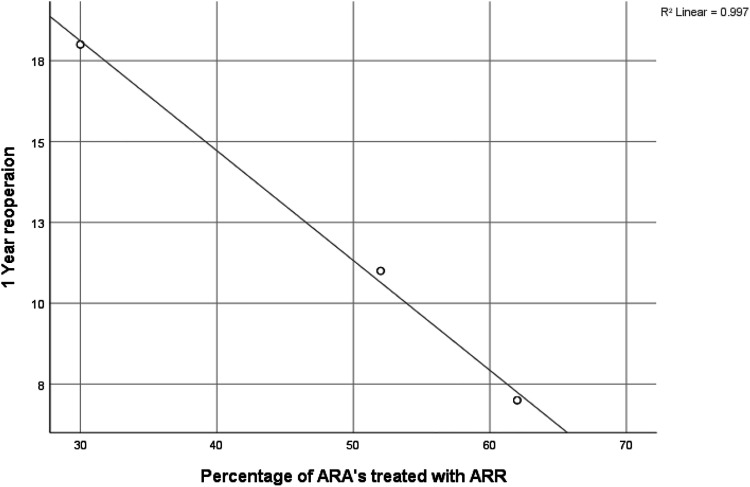
Simple scatter graph with regression line demonstrating institutional rates of reoperation given the percentage of ARA treated with ARR. Data extracted from Harris et al., Leontyev et al. and Yankah et al.^18,19^ (*R*^2^ = 0.997 r = 0.998).

Our post-hoc scatter graph provided an ecological perspective of the association between ARR and lower reoperation rates (Figure 4). Although only three studies could be included in this analysis, a strong linear relationship was seen (r = 0.998), suggesting hospitals with higher rates of ARR achieve lower rates of reoperation for ARA patients.^10,18,19^

## Discussion

The seminal findings from our meta-analysis were that ARA are associated with an increased risk of in-hospital mortality and late mortality. This may confirm that ARA remains a serious and aggressive complication of IE despite modern standards of care. However, due to limitations in the feasibility of experimental data, all studies included in our meta-analysis were observational, meaning that the overall quality of evidence was judged to be low and only cautious deductions could be made. Nevertheless, three of the four studies weighted in the analysis adjusted their mortality figures for cofounding variables. Additionally, the presence of an ARA was shown to have a reasonably large treatment effect on in-hospital mortality (OR 1.74 *p* = 0.005). Therefore, tentative causal inference can be made between ARA and elevated mortality.^13,14,17^

Our meta-analysis revealed an inconsistency in reoperation rates between institutions. In our previous study, we found significantly higher reoperation rates in patients with an ARA compared with NARA (RR = 2.71 95% CI: 1.18–6.24),^10^ a finding that was not consistent with studies published by Yang et al. and Said et al. (Figure 2). Different institutional preferences for ARR versus PR may explain this finding, as preceding reports have suggested that ARR can accomplish more comprehensive removal of infected tissue and reconstruction of cardiac morphology than PR, leading to reduced rates of re-infection and graft deterioration.^2,20^ Our post-hoc ecological analysis provides additional evidence to support this hypothesis, showing that institutions that perform more ARR achieve better reoperation figures. This is also coherent with the Chen et al. meta-analysis, which collated the results from studies that have compared the postoperative outcomes of PR versus ARR.^8^ Here, they showed that patients treated with an ARR were at a lower risk of reoperation at 1-year follow-up with no evidence of increased mortality. Therefore, there is growing evidence to suggest that ARR is associated with advantageous reoperation rates, a hypothesis that is also intuitive and backed by well-established surgical principles. Achieving lower reoperation rates carries significant benefits to patients in avoiding the serious complications which may follow a return to theatre while also having financial advantages for the healthcare system.

Chen et al.^8^ also reported that ARR was not a significant risk factor for increased early mortality. Evidence of subtle trends of ARR-associated mortality in observational studies is likely to be confounded by the higher risk population on which they were performed.^18,19^ This consideration does not explain lower reoperation rates observed in patients treated by ARR, so the disparity in reoperation rates can still be attributed to the operation itself. Therefore, we may be able to recommend a lower threshold for ARR in cases with less extensive damage to the aortic root in order to reduce reoperation rates for ARA patients. This change in practice could constitute a step forward in precise surgical decision-making for IE patients, bringing the management of this disease into the realm of evidence-based medicine. Nevertheless, more research is required in this area to establish the equity of ARR and PR in the treatment of ARA. Despite logistical difficulties and low incidence rates, future research efforts should focus on producing prospective cohort data with adequate statistical adjustment that will be able to refute or support the evidence outlined in this report.

### Limitations

Our meta-analysis has highlighted the limitations inherent to retrospective observational studies. Although some studies attempted to address this bias via a methodical approach to patient recruitment and utilisation of statistical models, this was not consistent in all studies and outcomes. Additionally, further inaccuracies could be introduced from unknown cofounders or insufficient sample size, which will limit the accuracy of those studies with statistical adjustment. A further limitation was a lack of consensus in outcome measures used in each study, this hindered data collection and the statistical power of our meta-analysis. Nevertheless, we believe that this study provides additional knowledge on the significance of ARA on postoperative outcomes and a new insight into surgical decision-making for this complication.

## Conclusion

This paper has observed an association between ARA and increased risk of early and late mortality despite modern standards of care. However, the causal relationship between ARA and mortality may be subtle as confounding factors were not adequately addressed by statistical models used in previous research. Using an ecological approach, we also add to evidence that the ARR technique is associated with an advantageous postoperative profile compared with PR. Clearly, more research is needed in this area with larger numbers but based on our findings and other published literature, ARR could be viewed as having a favourable postoperative reoperation rate.
